# Telemedicine in neurology: advances and possibilities

**DOI:** 10.1590/0004-282X-ANP-2022-S127

**Published:** 2022-08-12

**Authors:** Emanuelle Roberta da Silva Aquino, Soraya Camargo Ito Suffert

**Affiliations:** 1 Universidade de São Paulo, Faculdade de Medicina, Hospital das Clínicas, São Paulo, SP, Brazil.; 2 Hospital Sírio-Libanês, São Paulo, SP, Brazil.

**Keywords:** Telemedicine, Neurology, Remote Consultation, COVID-19, Delivery of health care, Telemedicina, Neurologia, Consulta Remota, COVID 19, Atenção à saúde

## Abstract

**Background:**

Telemedicine develops from technology that offers opportunities for knowledge transfer and information sharing and allows the provision of health services at a distance.

**Objective:**

To evaluate the number of publications on teleneurology in the last two decades in PubMed and the available evidence on the use of this technology in neurological clinical conditions.

**Methods:**

A quantitative assessment of publications related to telemedicine and neurology in the last two decades. A search was performed on the PubMed database for the descriptors ("Telemedicine"[Mesh]) AND "Neurology"[Mesh]). A review of the articles retrieved on the topic was carried out to evaluate the innovation processes used and applications in various clinical conditions involving teleneurology.

**Results:**

The search performed on March 14th 2022 resulted in 229 publications involving the topic of telemedicine and neurology between 1999 and 2022. Since 2000, there has been an increase in publications related to this topic, with a peak of 71 articles published in 2020, the year in which the World Health Organization defined the COVID-19 pandemic status.

**Conclusion:**

In the last two decades, teleneurology has been developing through the expansion of technological resources and the COVID-19 pandemic intensified this process. Different modalities of teleneurology are studied in several neurology subfields and include teleconsultation (between healthcare professionals or between healthcare professionals and patients), telerehabilitation, telemonitoring and tele-education. The advances achieved by teleneurology in this period encouraged technological innovations and health processes that developed opportunities to improve the care provided in a mechanism of constant evolution.

## RESUMO

**Antecedentes:**

A telemedicina se desenvolve a partir da tecnologia que permite a transferência de conhecimento e o compartilhamento de informações, possibilitando a prestação de serviços de saúde à distância.

**Objetivo:**

Avaliar o número de publicações sobre teleneurologia nas últimas duas décadas no PubMed e as evidências disponíveis sobre o uso desta tecnologia em diferentes quadros clínicos neurológicos.

**Métodos:**

Avaliação quantitativa das publicações relacionadas à telemedicina e neurologia nas últimas duas décadas. O levantamento dos artigos ocorreu a partir de busca no PubMed com os descritores (“Telemedicine”[Mesh]) AND “Neurology” [Mesh]). Foi realizada uma revisão dos artigos relacionados ao tema para avaliar os processos de inovação utilizados e aplicações em diversas condições clínicas envolvendo a teleneurologia.

**Resultados:**

A busca realizada no PubMed em 14 de março de 2022 resultou em 229 publicações envolvendo o tema telemedicina e neurologia entre 1999 e 2022. Desde 2000, houve um aumento de publicações relacionadas a esse tema, com um pico de 71 artigos publicados em 2020, ano em que a Organização Mundial da Saúde definiu o status de pandemia da COVID-19.

**Conclusão:**

Nas últimas duas décadas, a teleneurologia vem se desenvolvendo com a expansão dos recursos tecnológicos e a pandemia de COVID-19 intensificou esse processo. São estudadas diferentes modalidades de telemedicina em diversas áreas da neurologia, incluindo teleconsulta e teleconsultoria, telereabilitação, telemonitorização e teleducação. Os avanços alcançados pela teleneurologia nesse período estimularam inovações tecnológicas e processos de saúde que criaram oportunidades para melhorar a assistência prestada em um mecanismo de constante evolução.

## INTRODUCTION

Telemedicine in neurology has developed with the advent of technology, which provided an opportunity for knowledge transfer and information sharing. The applications of technology enable patient care, teaching and training of professionals and research development^1^. Telemedicine allows the provision of health services at a distance using technologies for connectivity, including telephone and internet, and may involve teleconsultation, teleconference, or even tele-education. Contact can be initiated by healthcare professionals or patients and can be synchronous (in real-time) or asynchronous (store and forward)^2^.

In December 2019, a new type of coronavirus, now known as SARS-CoV-2 (), was identified in China. SARS-CoV-2 infection (COVID 19) caused severe respiratory conditions and was associated with ICU admission and high mortality^3^. On March 11 2020 the World Health Organization characterized COVID-19 as a pandemic^4^. The pandemic has had a major socioeconomic impact and caused collapses in health systems around the world. Social distancing and the lockdown promoted a change in the offer of health care and the development of telemedicine. The COVID-19 pandemic highlights how neurological care was provided and how the mode of delivery needed to adapt quickly during this period, and how technology was fundamental in this process^5^. 

The aim of this study was to evaluate the number of publications on telemedicine in neurology in the last two decades in PubMed and the available evidence on the use of this technology in neurological clinical conditions.

## METHODS

This study carried out a quantitative survey of publications related to telemedicine and neurology in the last two decades. The survey of articles consisted e of a PubMed search on telemedicine in neurology in the last two decades with the descriptors ("Telemedicine"[Mesh]) AND "Neurology"[Mesh]. For the proper identification of publications/year, articles were classified by publication date as described in Table 1.

**Table 1.** Number of publications / year.

Year	Publications
1999	1
2000	5
2001	2
2002	4
2003	4
2004	4
2005	8
2006	2
2007	2
2008	4
2009	6
2010	6
2011	4
2012	4
2013	7
2014	7
2015	8
2016	7
2017	12
2018	15
2019	9
2020	71
2021	32
2022	5
Total	229

A review of the articles related to the topic was carried out to evaluate the innovation processes used and applications in various clinical conditions involving teleneurology.

This study was not submitted for approval by the Research Ethics Committee, as it is a non-systematic literature review to assess the development of telemedicine in neurology.

## RESULTS

**Figure 1.**
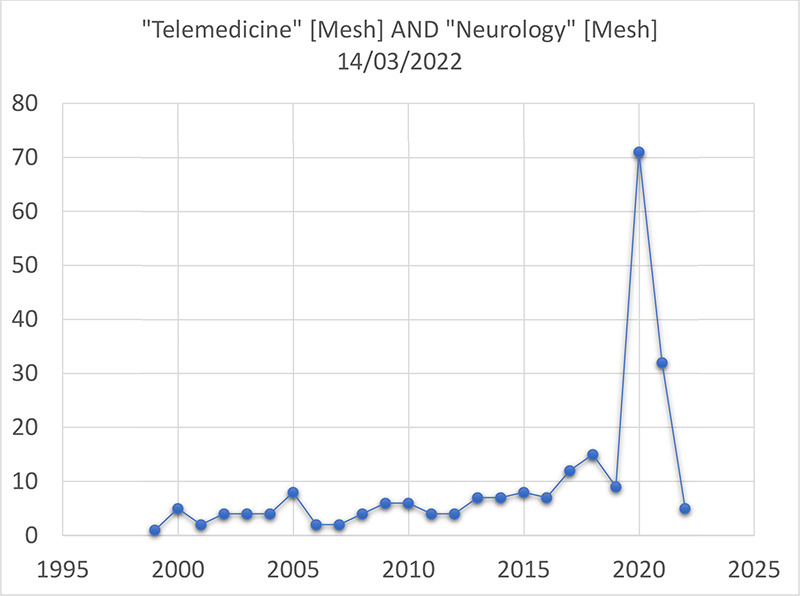
Number of publications in PubMed in the last 20 years.

The search performed on March 14, 2022 resulted in 229 publications involving the topic of telemedicine and neurology between 1999 and 2022. Since 2000 there has been an increase in publications related to this topic, with a peak of 71 articles published in 2020, the year when the World Health Organization declared COVID-19 a pandemic. The year 2021 presented a reduction in the number of publications, reaching 32, but maintaining a higher level of publications compared to the last 2 decades. The results are presented in Table 1 and in Figure 1.

## DISCUSSION

The emergence and consolidation of teleneurology is closely linked to the evidence for the benefit of stroke reperfusion therapies published in the 1990s^6^. The perception that every minute of delay in thrombolysis decreases the effectiveness of treatment and increases the risk of complications is promoted with the slogan “time is brain”^7^. In this context, where every effort is made to reduce the time between the onset of symptoms and the start of reperfusion therapy, telestroke allows remote specialists to indicate thrombolysis through information provided by the hospital physician, close to the patient^8^.

During the last two decades, the telestroke concept has spread around the world and been refined. Mobile stroke units were developed to optimize pre-hospital care. They are ambulances specially equipped with computed tomography, a point-of-care laboratory for blood analysis, medication and an interdisciplinary team. Telemedicine allows the support of a stroke specialist and, if indicated, thrombolysis can be started in the ambulance, before arrival at the hospital^9^.

Scientific evidence shows that stroke treatment has benefited from the digital health revolution. The use of teleneurology for stroke has significantly impacted the acute treatment of cerebrovascular diseases in many areas^10^.

Several experiences and a few randomized trials have been published on remote consultation between neurologists and patients with chronic neurological diseases. Patients suffering from headache were evaluated and studies showed that the use of telemedicine was not inferior to face-to-face care in terms of reducing attacks and recognizing secondary headaches^11^ ^-^ ^13^.

Evidence in the literature demonstrates that telemedicine care, through video or telephone interviews, is suitable for people with epilepsy. Outpatient epilepsy follow-up consultations depend essentially on phenomenological interviews, adherence to treatment and counseling, and not on physical examination. Studies have shown similar patterns of seizure control and medication adherence among patients with face-to-face and remote care^14^ ^,^ ^15^.

An important aspect evaluated in studies with telemedicine and cognitive impairment includes the diagnostic reliability of the administration of cognitive tests, such as the Montreal Cognitive Assessment (MoCA) and the Mini-Mental State Examination, showing that scores obtained remotely and face-to-face are comparable^16^ ^-^ ^19^.

Other studies indicate that telemedicine is a useful tool on the management of patients with dementia^20^ ^-^ ^22^, movement disorders^23^ ^,^ ^24^, including deep brain stimulation (DBS)^25^ ^,^ ^26^ follow-up, and in the attention of their caregivers^27^. 

The neurological examination involved in neuromuscular diseases has some items that are difficult to access by telemedicine, such as reflexes and vibratory sense. The same happens with vestibular disorders, with positional maneuvers, for example^28^. Before the COVID-19 pandemic, remote consultation in these areas was less consolidated. In the last two years, however, experience with this type of care has increased and telemedicine has been encouraged^29^. Current evidence indicates that telemedicine is a potential tool to be used as a complement to face-to face consultations in numerous areas of neurology^30^.

The development of telemedicine in neurology for chronic diseases is mainly motivated by the difficulty of access to the specialists in some regions. In several countries, there is a greater supply of specialists in capitals or tertiary services, which makes access difficult for rural populations or locations with greater distances from large centers. Access to care is also limited by patients' disabilities, as many neurologic conditions impair mobility and driving abilities. 

Telemedicine can have a positive impact on ensuring timely neurological care in inpatient or outpatient across different healthcare systems. The impact of the distance of access to the neurologist and the use of real-time video to promote timely access was the subject of the cohort study developed by Craig et al. in 2004. The study highlighted the difficulty of accessing specialist neurology consultations at two rural hospitals in Northern Ireland, with general physicians providing local care and referring patients to specialists when needed. Offering an early neurological consultation through real-time video was an alternative to promote access in a shorter waiting time for the neurologist with reduced hospitalization time^31^. 

 Reducing the waiting time for consultation with the neurologist was also the subject of the retrospective cohort by Constanzo et al. in 2020, involving analysis of primary and secondary care referrals of 8269 patients to neurology. Waiting time for the first consultation was 60% shorter for patients enrolled in the Teleneurology program in Chile, a country with great distances from some locations to access the capital, Santiago, the city with the highest concentration of neurologists^32^.

A program that allows primary care providers (PCP) to maintain direct contact with neurologists (eConsult service) in Canada has reduced the need for specialist consultation by more than 30%. The neurologist's answers to PCP questions can guide the management of patients with neurological complaints in primary care, which reduces the waiting time for patients who need a formal consultation with a neurologist^33^.

There is evidence showing benefits of telerehabilitation specially in patients after stroke^34^ ^-^ ^36^, but also in other neurological conditions, such as Parkinson disease^37^, vestibular disorders^38^ and multiple sclerosis^39^. Telerehabilitation is characterized not only by video contact between patient and physical therapist, but can include virtual reality and applications for self-managed exercises^36^ ^,^ ^38^, without therapist guidance. Telemedicine is useful in improving access to rehabilitation therapy and reducing travel time and costs.

Telemonitoring is already a reality and an increasingly promising area. It is possible to use portable vital signs measurement devices^40^, wearable sensors and smartphone apps to detect information and send to the doctor synchronously, 24 hours a day. It helps in the diagnosis, the assessment of the severity of neurological diseases and supports clinical decision making. Smartphones can capture voice and tremor and, and through machine learning, we can monitor or even diagnose Parkinson’s disease^41^ ^-^ ^43^. Assessing balance and risk of falls using a validated smartphone app can increase safety and reduce the number of falls in older adults^44^.

In the near future, a set of quantitative measures derived from wearables or apps could be considered digital biomarkers of a disease, contributing to early diagnosis, stratification of subgroups and prediction of treatment outcomes^45^.

 In addition to the application of telemedicine among healthcare professionals and between healthcare professionals and patients, teleneurology is also useful in education. An experience has shown that supervising neurology training with robot^46^ telepresence is feasible and remote consultations can be more comfortable for students and patients for teaching anamnesis and neurological diagnosis^47^.

The COVID-19 pandemic, with the recommendation of social distancing and the implementation of lockdown in several countries, was an important factor in the development of telemedicine to guarantee access to the first consultation and maintenance of patient follow-up. Ganapathy highlighted in its descriptive study in 2020, about telemedicine and neurological practice in the period of COVID-19, that real-time video synchronous queries or asynchronous digital store-and-forward services can be used. The latter includes text messages, WhatsApp communication and email. Remote monitoring of patient data can include viewing images and neurophysiological parameters^5^.

 The pandemic has certainly made Digital Health a necessity and no longer an exception. Technological advances that seemed distant possibilities and futuristic concepts came to fruition in a short period of time due to the needs imposed by the COVID-19 pandemic^10^.

### Teleneurology in Brazil

There are few Brazilian publications about teleneurology. A recent survey^30^ showed that before the pandemic only 18.5% of Brazilian neurologists worked with telemedicine and 31.7% studied telemedicine, while 63.6% reported working with telemedicine during the first year of pandemic^30^.

Telestroke is a reality in some Brazilian cities, but it is necessary to improve stroke care in all regions of the country, and the implementation of telemedicine support is recommended for hospitals without neurologists available 24 hours a day^48^ ^,^ ^49^. 

Several medical specialties have been included in government telemedicine programs with the aim of supporting primary care physicians and optimizing referrals for each specialty^50^ ^,^ ^51^. Mantese et al. evaluated 1,687 teleconsultations on patients with neurological complaints and showed that telehealth support could avoid 29% of referrals for neurology. The main reason for teleconsultation was epilepsy or seizure (25%), followed by headache (20%), stroke (10%), tremor or parkinsonism (7%) and cognitive disorders (6%)^52^.

In Brazil, some telemedicine procedures, such as remote consultations, were not regulated until ordinance 467 of the Ministry of Health^53^ and Law 13.989, of April 2020^54^, during the COVID-19 pandemic. After regulation, public and private health services organized remote consultations and several studies are now being submitted for publication. A descriptive study on telehealth in Recife during the COVID-19 pandemic included 126 remote neurological consultations and showed that in 68 the patient did not need to be referred to specialized face-to-face care after the consultation, with follow-up possible in primary care^55^. The authors are aware of different initiatives in teleneurology during the pandemic and we believe that Brazilian publications on telemedicine will increase significantly soon.

In conclusion, in the last two decades, teleneurology has been developing through the expansion of technological resources. The COVID-19 pandemic intensified this process and allowed for greater use of teleneurology to assist patients and to discuss cases between doctors at different levels of health care. Different modalities of teleneurology are studied in several neurology subfields and include teleconsultation (between healthcare professionals or between healthcare professional and patient), telerehabilitation, telemonitoring, tele-education.

The last two years have certainly made Digital Health a necessity and no longer an exception. Technological advances that seemed distant possibilities and futuristic concepts came to fruition in a short period of time due to the needs imposed by the pandemic. The advances achieved by teleneurology in this period encouraged technological innovations and health processes that developed opportunities to improve the care provided in a mechanism of constant evolution.
